# “The communication I had with him back then is still stuck in my mind.” Bereaved families of cancer patients’ experiences for end-of-life communication

**DOI:** 10.1007/s00520-023-07753-z

**Published:** 2023-04-18

**Authors:** Heejung Jeon, Sanghee Kim, Ilhak Lee

**Affiliations:** 1 Department of Nursing, Graduate School, Yonsei University, Seoul, Republic of Korea; 2 College of Nursing and Mo-Im Kim Nursing Research Institute, Yonsei University, Seoul, Republic of Korea; 3 Division of Medical Law and Ethics, Department of Medical Humanities and Social Sciences, Yonsei University College of Medicine, Asian Institute for Bioethics and Health Law, 50-1 Yonsei-Ro, Seodaemun-Gu, Seoul, Republic of Korea

**Keywords:** Cancer, Bereavement, Communication, End-of-life, Family, Qualitative research

## Abstract

**Purpose:**

Communication with family members is important to end-of-life care for patients with cancer. It is an interactive engagement between terminally-ill cancer patients and their families through which they expand their mutual understanding to cope with losses and find meaning in death. This study aimed to describe the experiences of end-of-life communication between patients with cancer and their family members in South Korea.

**Methods:**

This is a qualitative descriptive study using in-depth semi-structured interviews. Ten bereaved family members with end-of-life communication experience with terminal cancer patients were recruited through purposive sampling. Data were analyzed using qualitative content analysis.

**Results:**

A total of 29 constructed meanings, 11 sub-categories, and the following 3 categories were derived: “Offering a space for patients to reminisce and reflect,” “Building a bond,” and “Reflections on what we need.” End-of-life communication primarily centered on the patients, with families struggling to share their stories with them. Although the families coped well, they also regretted the lack of meaningful communication with the patients, indicating a need for support to facilitate effective end-of-life communication.

**Conclusion:**

The study highlighted concrete communication for finding meaning at the end-of-life for cancer patients and their families. We found that the families have the potential to communicate appropriately to cope with the patients’ end-of-life. Nevertheless, end-of-life presents a unique challenge in which families require adequate support. Given the increasing number of patients and families dealing with end-of-life care in hospitals, healthcare providers should be mindful of their needs and help them cope effectively.

**Supplementary Information:**

The online version contains supplementary material available at 10.1007/s00520-023-07753-z.

## Introduction

The increase in deaths due to cancer and an aging population are issues that are attracting attention worldwide [1, 2]. In South Korea, chronic diseases have increased among aging people, and cancer as the number one cause of death has steadily increased since 1983. Hospitals, the places of death, have had the highest rate since 2009 [3]. These social changes necessitate the comprehensive development of end-of-life care [4].

Although curing cancer is undoubtedly important, it is equally crucial to provide appropriate end-of-life care to terminally ill patients to ensure they experience a sense of dignity during their remaining life. The term “end-of-life” encompasses multiple definitions [5], referring to the period from the point of terminal diagnosis to death. This period is critical for patients to reflect on their lives and for their families to prepare for the eventual loss of their loved one.

As family members practically and emotionally support patients [6], healthcare providers should be mindful of their presence and its potential impact during the end-of-life process [7], in which, communication between a dying patient and their family is important. This mutual engagement helps patients and families understand each other and cope with terminal illness, thus offering a means of care for both parties while they find meaning in death. Through end-of-life communication, patients and their families can find closure and peace, enabling them to face the end with acceptance and dignity.

SARS-CoV2 has affected how families provide care, causing negative experiences in communication, especially for end-of-life patients [8, 9]. Studies related to end-of-life care emphasized patient-family communication as an important process that helps patients prepare for death and their families cope with bereavement [10, 11]. However, studies in this field are currently limited, and no qualitative studies have been conducted in South Korea.

Healthcare providers have a responsibility of providing comprehensive care encompassing not only physical care, but also psychological, social, and emotional care [12]. With the increasing number of patients receiving end-of-life care in hospitals, healthcare providers’ awareness and appreciation of the emotional and social experiences of patients and their families during this period has become important. To provide the best possible end-of-life care, healthcare providers should deeply understand how families and patients make intersubjective sense of impending death and confront the reality of loved ones dying. Therefore, it is necessary to examine the end-of-life experiences of patients from the perspectives of their families. As families often serve as primary caregivers, they play an important role in the patients’ experiences. This study aimed to examine the end-of-life communication experiences of bereaved families of cancer patients, providing valuable insights for end-of-life care.

## Methods

This is a qualitative descriptive study that aimed to collect rich and descriptive data with minimized interpretation to achieve a common understanding and agreement among people regarding a specific phenomenon [13]. Qualitative descriptive studies are considered a suitable research method in the healthcare field. The study was based on a constructivist approach, which assumes that truth or meaning is constructed by individuals through their experiences [14]. With this philosophical underpinning, data were collected through a 1:1 in-depth interview to achieve a rich description. And qualitative content analysis, as described by Elo and Kyngäs, was used to analyze the data [15].

### Study participants

The participants were the bereaved family members of terminal cancer patients with end-of-life communication experiences. We recruited the participants through purposive sampling to identify information-rich cases related to the research context. For this, recruitment notices were posted at five hospitals and two non-profit support organizations for bereaved families. Families who were interested in participating contacted us by e-mail or phone. We screened potential participants to ensure that they met the eligibility criteria for the study. The inclusion criteria were: (i) adults over the age of 19, (ii) family members of patients with cancer, (iii) family members of patients who died in hospitals, and (iv) family members who had been bereaved for fewer than five years. Family members of patients who died of a non-cancer cause were excluded.

### Data collection

In-depth interviews, which followed a semi-structured guide, were conducted at the participants’ residences to establish a comfortable environment. The guide was refined after two experts’ review and two pilot tests as shown in Table 1. The interview was conducted between August and September 2021 for about 90–120 min for each individual. The participants were interviewed by the first author, and all interviews were audio recorded. There were no withdrawals from the study.

**Table 1:** Semi-structured interview guide

< Main questions >
∙ The topics of communication when the patient was diagnosed with the terminal cancer
∙ The nature of conversation with the patient during hospitalization
∙ The nature of conversation when the patient was approaching death

### Data analysis

Each interview was directly transcribed and analyzed on the day it was conducted to guide the next interview. A qualitative content analysis with an inductive approach, commonly used in qualitative descriptive studies, was performed on the transcripts [15]. First, we reviewed the transcripts multiple times to gain insight, and selected the unit of analysis to identify significant content. Second, each researcher conducted open coding to identify meaningful statements, which all researchers repeatedly evaluated to arrive at a final set of meaningful statements. Third, statements with similar meanings were grouped and reduced to higher-level categories, including sub-categories and main categories, for the identification of common themes. Finally, the categories were formulated through abstraction.

### Ethics

The Institutional Review Board of the Yonsei University Health System, Severance Hospital, approved the study (No. 4–2021-0582).

### Rigor

We adopted Lincoln and Guba’s four dimensions—credibility, transferability, dependability, and confirmability—to guide our research approach [16]. To enhance credibility, we performed member-checking with two participants and utilized peer-debriefing. To ensure transferability, we described our research context comprehensively and in detail. To ensure dependability, we followed a rigorous and systematic approach to data analysis. To ensure confirmability, we minimized biases by keeping field notes and reflecting on the entire research process.

## Results

Characteristics of the 10 participants are shown in Table 2. The content analysis resulted in 29 meanings and 11 sub-categories. Finally, three categories were identified as follows: “Offering a space for patients to reminisce and reflect,” “Building a bond,” and “Reflections on what we need.”

**Table 2:** Characteristics of participants

	Gender /Age	Relationship with patient	Main caregiver	Type of hospital	Religion	Year of bereavement
#1	F/32	Child	N	General hospital	Christianity	2020
#2	F/78	Spouse	Y	General hospital	Christianity	2019
#3	M/43	Child	N	Hospice	Christianity	2018
#4	F/54	Child	Y	Hospice	Buddhism	2020
#5	F/69	Spouse	Y	Hospice	Christianity	2017
#6	M/44	Sibling	N	Hospice	Atheism	2020
#7	F/70	Spouse	Y	General hospital	Christianity	2016
#8	F/31	Sibling	N	General hospital	Catholicism	2016
#9	F/68	Child	Y	Hospice	Christianity	2020
#10	F/51	Spouse	Y	Hospice	Buddhism	2020

### Category 1. Offering a space for patients to reminisce and reflect

#### Embracing patients’ mixed feelings

The patients experienced changes in their physical appearance as the disease progressed. They expressed their feelings to their families, sometimes avoiding the mention of the disease.

*“When he finds it difficult to accept, he wonders why he is suffering from this disease. He said, ‘I lived a difficult life, but why should I be sick?’” (Participant #10)*

The patients knew about the changes in their life as a result of their illness, and showed their negative feelings—depression and distress—about their situation.

*“The weather is so nice in spring, but he’s only lying in the hospital. Such weather makes him feel worse. He said, ‘If the weather is good, I’d like to go out and play, but I can’t. So, I’d rather have a rainy day.’” (Participant #8)*

#### Helping patients plan for the future

The patients felt annoyed talking about the things left undone. Their families encouraged them to imagine and plan their future to avoid frustration.

*“He’s about to die, but I didn’t want to think he had no future. So, I asked, ‘what do you want to do in the future?’ I let him talk as much as he could about things he couldn’t do [before] because he was busy.” (Participant #10)*

#### Listening to patients’ reflection on their lives

The patients reflected on their past, recalling specific memories related to their childhood and adulthood.

*“In about three months, he began to reflect on the life he has lived since he was born. We talked about what happened at school, at work, etc., and about our happiest time in [our] lives.” (Participant #5)*

The patients identified unresolved conflicts and forgave the concerned people through communication with their families instead of directly communicating with those people.

*“His mother has hurt him a lot. So, he cried a lot; he was deeply hurt. But this is what he said, ‘Don’t hate my mother too much, I’m sorry, but please don’t hate her.’” (Participant #10)*

### Category 2. Building a bond

#### Expressing one’s emotions

The patients and their families expressed their positive and negative feelings to each other. Positive feelings were identified as “love” and “gratitude.” The families believed that conversations about affection and love during this period were more meaningful than usual.

*“What I remember is that she called out my name and said, ‘I love you.’ I think it was something she really wanted to tell me even though it was so difficult.” (Participant #6)*

Negative emotions expressed were “fear,” “being sorry,” and “feeling upset.” The patients and their families feared that the patients may die—they were afraid to say “goodbye” to each other in the face of death. The patients felt sorry for their families who cared for them and were worried about their burden of care.

*“He said, ‘I don’t want to be sick either. I’m sorry I’m sick.’” (Participant #8)*

#### Talking about the health state and plan

The level of communication regarding the health status and care plan between the patients and their families varied from the active case to the passive one. In the active case, the patients tried to reassure their families, who tried to listen to the patients’ concerns with more interest. The patients and their families shared their thoughts on the treatment plan and the families tried to respect the patients’ opinions.

*“The doctor explained to him about forgoing life-sustaining treatment, and he agreed with it. I wanted to save him for one more day. But he told me, ‘Even if things go wrong, don’t try to save me any longer.’” (Participant #10)*

In the passive case, the families knew about the patients’ disease status, but they feared to tell the patients about it, despite the potential benefits of helping the patients become prepared for the end. They were cautious about being honest, but eventually found courage.

*“I didn’t know how to tell my mom. … After thinking about it for a long time, I finally asked, ‘Mom, do you know what’s wrong with you? It’s pancreatic cancer.’ She replied, ‘I know.’ I was worried a lot that she would be shocked if I told her, but she was calm.” (Participant #4)*

Some families were apprehensive about openly discussing their loved one’s health status and plan. The families chose to offer words of hope to their loved ones while recognizing the inevitability of the end-of-life because they feared that honest communication would damage the patients’ will to live. However, only offering impossible hope to the patients emotionally distressed the families, as they knew that the end was near.

*“Eventually, I thought that there was no way to cheer my brother up with words. I always said, ‘You can do it.’ I ended up only talking about impossible things.” (Participant #8)*

#### Saying “Goodbye”

The patients and their families said “goodbye” to each other individually. Sometimes, the patients said "goodbye” to their families when they accepted their impending death. The families stayed by the patients’ sides until the end, offering prayers and comfort during their final moments. As death approached, the patients could not respond or provide feedback, limiting communication to one way. Nevertheless, the families provided unwavering support, offered comfort, said final goodbyes.

*“I said goodbye to my brother. I don’t know if he heard it. But a bit later his eyes seemed to be filled with tears. So, I whispered to him that he should not cry.” (Participant #8)*

#### Staying connected with the patient after bereavement

The families stated that they did not want to accept that their loved ones were dying while they were battling the disease. They only acknowledged that the patients had been in the dying phase. Despite being heartbroken at this time, the families remembered their communication with the patients as meaningful event. The families fondly recalled the moments of end-of-life communication. The families kept their loved ones’ memories alive and maintained a sense of connection with them even after bereavement. The families continued to honor their loved ones by recalling these essential memories of end-of-life communication that occupies a special place in their hearts.

*“Although the contents of the conversation may be the same as that of our daily conversations, the communication I had with him back then is still stuck in my mind.” (Participant #1)*

#### Reflecting on families’ usual communication patterns

The patients who had good ties with their families could communicate sufficiently at the end of their life. However, those who had bad relationships experienced difficulties. The families with sufficient information and knowledge on death applied their experiences to the process of end-of-life communication with the patients, which facilitated their communication.

### Category 3. Reflections on what we need

#### Regret

The families regretted not having a chance to talk about what they wanted to discuss as they focused more on caregiving. The families found it difficult to express love actively because they feared that the patients’ desire to live would be discouraged. Consequently, the families regretted that they had to express their love passively.

*“When my brother said he loved me, I thought I’d make him nervous if I reacted differently; so, I just didn’t respond. I regret that.” (Participant #8)*

Further, when the families did not honestly inform the patients of their physical condition, the families regretted not providing the patients a chance to prepare for their death.

#### Restrictions due to SARS-CoV2

With the SARS-CoV2 outbreak, hospitals started restricting visits to prevent the spread of the disease, which made communication between the patients and their families difficult. The limited time they could spend together made the family members feel anxious. Particularly, the families said tearfully that the patients must have felt lonely. The families considered non-verbal communication important at the last moment of life, regretting that they could not hold the patients’ hands or be by their side because of visitation restrictions.

*“I could not see his face at the end because of SARS-CoV2. I think he couldn’t have talked because of his condition and he would have felt comfortable if I stayed with him by just holding his hand.” (Participant #1)*

Amid the strict hospital visitation restrictions imposed during the COVID-19 pandemic, the families of patients who were discharged but required readmission later because of worsening conditions prioritized hospitals with more lenient visitation policies. Overall, the families said that the medical environment was not conducive to spending meaningful time with the patients at end-of-life and called for improvements in end-of-life care.

#### Need support of healthcare providers

Families faced difficulties communicating with patients during the end-of-life because of insufficient information provided by medical staff. The families desired specific and adequate information regarding diseases, prognosis, and symptoms, along with a supportive attitude from medical staff. When the patients received such support, they felt respected and could prepare for end-of-life with their families, which facilitated mutual communication and strengthened their relationship.

*“The patient was sensitive to the way the medical staff speaks and behaves. He was under stress about that. The doctor spoke very nicely. Thanks to him, my husband felt that he was alive and tried to have a good time with us. I thought the role of medical staff in our communication was important.” (Participant #10)*

## Discussion

This study examined the end-of-life communication experiences of the bereaved families of cancer patients. The findings showed that patients with cancer and their families communicated specifically about approaching death at the end-of-life. During end-of-life communication, families provided patients with opportunities for reflection and formed stronger bonds with them. Additionally, following the bereavement, families reflected on their needs during end-of-life communication. We identified the key characteristics of end-of-life communication and practical implications.

### Characteristics of end-of-life communication

First, the families experienced an improvement in their relationship with the patients through end-of-life communication. The mutual communication strengthened familial solidarity and bonds. The findings of our study align with those of previous research [17–20], which emphasized the importance of families providing time for reflection to patients and engaging in meaningful communication to strengthen their relationship. Adding our results to this body of literature reinforces the theoretical validity of the contents of end-of-life communication between patients and their families.

Second, the families experienced difficulties in communicating with the patients, although they recognized it as an important activity. During the end-of-life situation, families prioritized patients’ well-being in all respects and had difficulty in honest communication because of psychological barriers, such as fear [21]. Reflecting the cultural norms prevalent in South Korea [22], the families exhibited a passive and rigid communication style, which inhibited active expressions of love. This defensive attitude in their communication lead to difficulties in selecting topics to talk about in the end-of-life period [23–25]. The families lacked confidence in their ability to communicate effectively even if given the chance to go back: *“Even if I go back, would I be able to admit the situation and tell what I want to say well?” (Participant #8)*. In end-of-life care, honest communication between the patients and their families is considered important [20, 26]. Many patients and their families still consider it important to talk about death before the final day [27]. Differences in culture, race, and the like should be considered for establishing strategies and methods for effective end-of-life communication [18, 28, 29].

Finally, achieving a balance in dialog is important in end-of-life communication. Although the patients were typically the main speakers, they often required the assistance of their families to communicate effectively. In such situations, families were responsible for listening and guiding the conversation. However, families often struggled to express what they wanted to say until the final day, and they regretted this after the patients’ death. Reflectivity around communication—reflecting on whether the message was properly delivered and why the patient responded so, and the like—occurred after the patient’s death.

### Implications for practice and policy

The “end-of-life” context should be considered for facilitating communication at that moment. An imbalance of dialogue in end-of-life communications has an important effect on the family’s bereavement [9, 30]. Healthcare experts who provide end-of-life care should consider the vulnerability of terminally-ill cancer patients and their families. These patients are those who have to be prepared for goodbyes, not those who recover through treatment; their families have to start their new normal life with a drastically changed reality after the patients’ death. Our research shows that they have the potential to communicate adequately for coping with the end-of-life. Nevertheless, the end-of-life period presents a unique challenge and requires adequate support. In particular, for those experiencing end-of-life communication difficulties, mediation by healthcare providers can help families focus on caring for dying patients and helping them prepare for death [28, 31]. To facilitate communication, the complex and diverse factors of patients and their families should be considered, and strategies derived from the perspective of “individuals” are crucial [7, 32–34]. By integrating the important insights on end-of-life communication derived from our research, healthcare providers can facilitate efficient access within a time-constrained medical setting. To achieve this, healthcare professionals should apply their expertise to identify the key challenges in patient-family communication and implement effective interventions [35]. They should understand the meaning and message of end-of-life communication and become sensitive to individuals’ need for a complete life as human beings, not as patients.

When unexpected situations occur, such as the COVID-19 pandemic, medical environments should be sensitive to the need for extra support. Although it is important for the family to be with a patient during the end-of-life [36, 37], restrictions on hospital visits because of COVID-19 interfered with the end-of-life communication between patients and their families [24, 38–41]. Despite these restrictions, the importance of family visits during end-of-life should not be overlooked [42–45]. During a public health crisis, patients at the end-of-life and their families should be able to have meaningful communications and say goodbye. To come up with safe and satisfactory support measures for end-of-life communication, an in-depth understanding of the communication experience during a public health crisis is required and quantitative and qualitative research on various approaches should be actively conducted.

## Limitations

This study sheds light on end-of-life communication between patients and families and offers insights into their relationship during this challenging period. However, limitations include interviewing only one family member, thus overlooking family dynamics. Future research should include all family members and examine their communication patterns. Additionally, further research should consider various healthcare experts’ views and experiences on patient-family interactions. This will provide a more comprehensive understanding of end-of-life communication and enable the improvement of support for patients and families during this difficult time.

## Conclusion

The COVID-19 pandemic was an important era to reflect on end-of-life communication between cancer patients and their families. This study provided a way for cancer patients and their families to communicate concretely about finding meaning at the end-of-life. Given the increasing number of patients receiving end-of-life care in hospitals, healthcare providers should acknowledge the potential for families to understand and navigate communication with patients in these situations. Cancer patients and their families face unique challenges during this time and need adequate support to cope. Therefore, healthcare providers should be proactive in providing appropriate assistance to patients and their families who may be struggling.

## Supplementary Information

Below is the link to the electronic supplementary material.

Supplementary file1 (DOCX 22.6 KB)

## Data Availability

Please contact the corresponding author.
