# Supplementary material for: “The communication I had with him back then is still stuck in my mind.” Bereaved families of cancer patients’ experiences for end-of-life communication
Source: Support Care Cancer. 2023 Apr 18;31(5):277. doi: 10.1007/s00520-023-07753-z (PMC10111311; doi:10.1007/s00520-023-07753-z)
Supplement: Supplementary file 1 — Supplementary file1 (DOCX 22.6 KB) [file 520_2023_7753_MOESM1_ESM.docx]

Supplementary Table. Interview categories

| **Formulated meanings** | **Sub-categories** | **Categories** |
| --- | --- | --- |
| Patients regret their situation. | Embracing patients’ mixed feelings | Offering a space for patients to reminisce and reflect |
| Patients explain to their families difficulties and discomfort they experience. |  |  |
| Patients explain to their families their fear and worries about their changing appearance. |  |  |
| Patients tell their families about their favorite things. | Helping patients plan for the future |  |
| Families initiated the communication so that patients can tell their stories. |  |  |
| Patients tell their families what they intended to do in the future if not for the sickness. |  |  |
| Patients tell their families about their life. | Listening to patients’ reflection on their lives |  |
| Patients identify and resolve conflicts they had with others through communication with their families. |  |  |
| Patients and their families express positive emotions to each other. | Expressing one’s emotions | Building a bond |
| Patients and their families express negative emotions to each other. |  |  |
| Patients and their families want to make each other feel comfortable. | Talking about the health state and plan |  |
| Patients and their families hope to be comforted through spiritual expectations. |  |  |
| Patients tell their families about their physical discomfort. |  |  |
| Patients and their families discussed their views on the treatment. |  |  |
| Families tell patients about the bad news regarding their health. |  |  |
| Patients tell their families what they hope for the well-being of their families. | Saying “Goodbye.” |  |
| Families say “goodbye” to patients. |  |  |
| Families remembered the conversation they had with the patients when the patients were dying. | Staying connected with the patient after bereavement |  |
| Families look back at that time from patients’ perspectives. |  |  |
| The usual relationship between patients and their families affects end-of-life communication. | Reflecting on families’ usual communication patterns |  |
| The experiences and personalities of patients and their families are reflected in the communication. |  |  |
| Families regret not forgiving and reconciling with patients. | Regret | Reflections on what we need |
| Families are sorry for not being able to say “goodbye” to patients. |  |  |
| Families regret expressing love passively to patients. |  |  |
| Families are sorry for not disclosing the truth about the disease to the patients. |  |  |
| Families had difficulty having non-verbal communication with patients because of the pandemic | Restrictions due to SARS-CoV2 |  |
| Families feel upset because of the limited medical and care support services the patients received. |  |  |
| Families had difficulty in communication at the end of life because of limited information. | Need support of healthcare providers |  |
| Families talk about the medical staff’s attitude when the patients were dying. |  |  |
